# Treatment of chronic heart failure in Germany: a retrospective database study

**DOI:** 10.1007/s00392-017-1138-6

**Published:** 2017-07-26

**Authors:** Stefan Störk, Renate Handrock, Josephine Jacob, Jochen Walker, Frederico Calado, Raquel Lahoz, Stephan Hupfer, Sven Klebs

**Affiliations:** 1 Comprehensive Heart Failure Centre Würzburg and Department of Internal Medicine I, University and University Hospital Würzburg, Am Schwarzenberg 15, 97078 Würzburg, Germany; 2 Novartis Pharma GmbH, Nuremberg, Germany; 3 Elsevier Health Analytics, Berlin, Germany; 4 Health Risk Institute, Berlin, Germany; 5 Novartis Pharma AG, Basel, Switzerland

**Keywords:** Treatment pattern, Cardiologist, Germany, Heart failure, Guidelines, Care pathway

## Abstract

**Background:**

Adherence to treatment guidelines affects outcomes in patients with chronic heart failure (HF). We investigated patient pathways and treatment patterns for HF in Germany.

**Methods:**

This retrospective study used anonymous healthcare claims data from the German Health Risk Institute on individuals with statutory health insurance. Patients with uninterrupted data from 1 January 2009 to 31 December 2013 or death (whichever occurred first), and ≥2 recorded HF-related diagnoses in 2011, were included. Patients with newly diagnosed HF were identified. Use of treatment patterns recommended by the European Society of Cardiology (2008) and German Nationale VersorgungsLeitlinien (2011) guidelines was evaluated.

**Results:**

Of 123,925 patients with HF, 21.3% were newly diagnosed. Overall, 63.2% of new HF diagnoses were made in the ambulatory setting; 61.6% of these were made by family practitioners and 14.8% by cardiologists. In the ambulatory setting, family practitioners were primarily responsible for treatment; specialists in internal medicine (70.3% cardiologists) were mainly responsible for performing HF-related technical diagnostics. One-fifth (20.9%) of patients received a New York Heart Association (NYHA) classification; 45.1% of these received a guideline-based treatment pattern. Application of the recommended treatment pattern decreased with advancing disease severity (NYHA class IV: 21.1% application) and older age (≥90 years: 28.3% application).

**Conclusions:**

Family practitioners play a key role in the diagnosis and initial treatment of HF in Germany. A substantial proportion of patients do not receive guideline-recommended pharmacotherapy. These findings should be reflected in the planning of national disease management programmes.

**Electronic supplementary material:**

The online version of this article (doi:10.1007/s00392-017-1138-6) contains supplementary material, which is available to authorized users.

## Introduction

In Germany, medical care for patients with chronic heart failure (HF) is provided by various healthcare professionals in different healthcare sectors, including hospital-based cardiologists, office-based cardiologists, and/or general practitioners (GPs) [1]. Usually, patients are seen by a primary care GP who refers them to a specialist when further input is required [2]. Ideally, treatment should be based on the European Society of Cardiology (ESC) guidelines for the diagnosis and treatment of acute and chronic HF [3], and/or the National Guideline on Chronic Heart Failure (Nationale VersorgungsLeitlinien Chronische Herzinsuffizienz, NVL) [4]. However, diagnostic approaches and treatment decisions and, importantly, the methods of communicating these, vary substantially across the different physician groups [5–7]. In addition, the availability of diagnostic tools differs between settings; for example, because GPs are usually not reimbursed for performing echocardiography [8] they rarely use this tool; however, echocardiography is used regularly by office-based cardiologists and hospital-based physicians [9]. Variations may also arise owing to the characteristics of patients per setting [10, 11]; for example, patients with severe HF and acute decompensation are more likely to be admitted to hospital, while elderly patients and individuals with less severe HF are frequently treated by GPs in collaboration with an office-based cardiologist [12].

Approximately, 50% of patients with HF have a reduced ejection fraction (rEF; defined as a left ventricular ejection fraction <35–40%) [3, 4]. Most clinical trials published after 1990 have targeted patients with HFrEF [3]. Thus, guidelines published by the ESC [3] and the NVL [4] provide recommendations mainly for the treatment of this patient population, based on the New York Heart Association (NYHA) classification. Guidelines available at the time of this study (ESC 2008 [13] and NVL 2011 [14]) both recommended the use of an angiotensin-converting enzyme (ACE) inhibitor [or angiotensin II receptor blocker (ARB) when an ACE inhibitor was not tolerated] in addition to a β-blocker in all patients with symptomatic HFrEF categorized as NYHA class II–IV [14]. Furthermore, diuretics were recommended for symptom relief if congestion and/or peripheral oedema was present. Both guidelines also recommended the use of a mineralocorticoid receptor antagonist (MRA) for patients with HF of NYHA class III–IV unless this was contraindicated or not tolerated. For patients with HF of NYHA class I, both guidelines recommended monotherapy with an ACE inhibitor or an ARB. There is currently no approved treatment specifically for HF with a preserved ejection fraction (HFpEF), which comprises about half of all cases of HF [15], although similar drug classes (diuretics, ACE inhibitors/ARBs, MRAs, and β-blockers, as well as calcium-channel blockers) may be given to control symptoms and/or blood pressure [3, 16].

Although the safety and efficacy of existing therapies have been demonstrated in clinical trials, little is known about the diagnosis and treatment patterns of HF in clinical practice in Germany. Recently, several observational studies investigating the treatment of patients with HF in Germany have been conducted, although these were on a small scale [2, 17, 18]. The aim of this large-scale analysis of a representative sample of patients with HF in Germany was twofold: (1) to describe where and how patients with newly diagnosed HF interact with the healthcare system; and (2) to evaluate the use of common treatment patterns recommended by the ESC and NVL guidelines for HF.

## Methods

### Study design and objectives

This was a retrospective healthcare claims study conducted using data obtained from the German Health Risk Institute (HRI) research database, which contains anonymized data from ~7 million individuals with statutory health insurance (SHI) collected between 2008 and 2013 [19]. Data within the database are provided mainly by company and guild health insurers. Of the 81.2 million inhabitants in Germany, 70.6 million have SHI, while the remaining individuals have private medical insurance [20]; based on this, it has been estimated that the database contains data on ~10% of the population with SHI in Germany.

The HRI database is updated on a monthly basis and provides a complete data set for each patient across all available health care sectors (ambulatory and hospital care; pharmaceuticals; medical aids and remedies; sick leave). Information on the utilization of services on an individual basis enables patient-level analysis of disease and treatment data. For this study, a subset of ~4 million individuals from the 7 million in the database, was selected based on the age and sex distribution of the German population as of 31 December 2011 [19].

Data collected in the HRI database include the following: patient demographics (comprising age, sex, region or place of residence, insurance start and end dates, and date of death, if applicable); indices of outpatient care, including International Classification of Diseases and Related Health Problems, Tenth Edition, German Modification (ICD-10-GM) codes; hospital admission and discharge dates; diagnoses/reasons for hospitalization; details of pharmacy prescriptions and dispensations, including dates and costs of prescriptions; and the speciality of the physician responsible for diagnosis (e.g., cardiology, primary care), performance of procedures (e.g., laboratory, radiology, echocardiography), and prescription of medication.

The first objective of this study was to describe the pathway of patients with newly diagnosed HF, in terms of how they interact with the healthcare system from diagnosis through to treatment. For this, data on the following were evaluated: (1) the healthcare sector in which a first diagnosis of HF was made; (2) the speciality of the physician responsible for making the first diagnosis; (3) the healthcare sectors with which patients had contact following diagnosis; and (4) the specialities of physicians responsible for treating these patients and performing technical diagnostics for them. The second objective was to determine the proportion of patients that received treatment consistent with two common treatment patterns during the two years after diagnosis. The definition of the two chosen patterns (Table 1) was based on treatment recommendations of the ESC 2008 [13] and the NVL 2011 [14] guidelines at the time of the study, and is explained in detail in the ‘Data analysis’ section below.

**Table 1:** Treatment regimens recommended for patients with heart failure with a reduced ejection fraction

Scenario	NYHA class	Minimum requirement
Strict pattern	NYHA I	ACE inhibitor or ARB
	NYHA II	(ACE inhibitor or ARB) and β-blocker and diuretic
	NYHA III–IV	(ACE inhibitor or ARB) and β-blocker and diuretic and MRA (spironolactone or eplerenone)
Less strict pattern	NYHA I	ACE inhibitor or ARB
	NYHA II	(ACE inhibitor or ARB) and β-blocker and diuretic
	NYHA III–IV	(ACE inhibitor or ARB) and β-blocker and diuretic

*ACE* angiotensin-converting enzyme, *ARB* angiotensin II receptor blocker, *MRA* mineralocorticoid receptor antagonist, *NYHA* New York Heart Association

### Study population

To be included in this analysis, patients had to have observable data available without interruption from 1 January 2009 to 31 December 2013 or death (whichever came first). Individuals who died before 1 January 2011 were excluded. Patients were required to have at least two documented HF-related diagnoses, according to ICD-10-GM codes, in either a hospital or an ambulatory setting during the identification period (1 January–31 December 2011). This population is described as the total population with HF (Table 2 shows the ICD-10-GM codes used to justify the inclusion of patients in this population). The quarter of the calendar year in which the first HF diagnosis occurred was defined as the index quarter. A subgroup of the total population who were newly diagnosed with HF was identified based on the absence of a documented HF-related diagnosis in the year before the index quarter. Differentiation between patients with HFrEF and HFpEF on the basis of ICD-10-GM codes was not possible.

**Table 2:** ICD-10-GM codes used to include patients in the total population with heart failure

ICD-10-GM code	Corresponding diagnosis
I50.0	Right ventricular failure
I50.00	Primary right ventricular failure
I50.01	Secondary right ventricular failure
I50.1	Left ventricular failure
I50.11	NYHA class I
I50.12	NYHA class II
I50.13	NYHA class III
I50.14	NYHA class IV
I50.19	NYHA class not specified
I50.9	Heart failure, unspecified
I11.0	Hypertensive heart disease with (congestive) heart failure
I13.0	Hypertensive heart and renal disease with (congestive) heart failure
I13.2	Hypertensive heart and renal disease with both (congestive) heart failure and renal failure

*ICD*-*10*-*GM* International Classification of Diseases and Related Health Problems, Tenth Edition, German Modification, *NYHA* New York Heart Association

When available, information on specific NYHA class was also derived from the ICD-10-GM code. Patients with more than one NYHA class documented were assigned the last NYHA class documented in 2011. Any patient without a specific NYHA diagnosis was assigned to an HF group named ‘other’.

All patient data in the HRI database were anonymized and reflect the routine treatment of individuals in daily life; therefore, patient consent and approval by an independent local ethics committee was not required.

### Data analysis

#### Pathway of patients with newly diagnosed HF

The patient pathway was assessed for the population of individuals with newly diagnosed HF. The number and proportion of patients newly diagnosed with HF were recorded at the index quarter by physician speciality (e.g., GP, internal medicine, obstetrics and gynaecology, paediatrics, psychiatry, neurology, or radiology) and hospital unit (e.g., cardiology, critical care, diagnostic imaging, accident and emergency, general surgery, gastroenterology, or ear, nose and throat), as well as by healthcare sector (ambulatory, hospital inpatient, or hospital outpatient). In addition, the number and proportion of patients with at least one visit to an office-based physician, or to hospital as an inpatient or outpatient, in the two years after the index quarter were recorded by quarter. Lastly, the number and proportion of patients being treated undergoing technical diagnostics by the various physician specialities or hospital units were recorded for two years after the index quarter.

#### Use of common guideline-recommended treatment regimens in all patients with HF

Medications were considered ‘HF-related’ if they matched a predefined list of substance classes commonly prescribed to patients with cardiovascular disorders (Online Resource 1). Medication was classified using the Anatomical Therapeutic Chemical Classification System codes taken from the Anatomical Therapeutic Chemical/Defined Daily Dose Index 2013 (German version) provided by the German Institute of Medical Documentation and Information. Procedures were retrieved using the codes of the German Operationen- und Prozedurenschlüssel.

All patients were assessed to ascertain whether, in the 2-year period after first diagnosis, they were receiving therapy consistent with two common treatment patterns outlined for HF of each NYHA class (see Table 1). The two patterns were based on the ESC 2008 [13] and NVL 2011 [14] guidelines for the treatment of HF. Treatment gaps of up to three months were permitted between prescriptions. The focus of this analysis was on the use of therapies known to reduce mortality. ACE inhibitors or ARBs were included in both patterns for all patients with HF of NYHA class I–IV, and β-blockers were included for all patients with HF of NYHA class II–IV. According to the respective guidelines, MRAs should have been considered in all patients with HF of NYHA class III–IV unless contraindicated or not tolerated (e.g., presence of gynaecomastia in men, hyperkalaemia, or renal dysfunction). It was not possible, however, to determine which patients had a contraindication to or did not tolerate MRAs. We, therefore, analysed the proportion of all patients receiving treatment consistent with two patterns in the two years after diagnosis: the so-called ‘strict’ pattern included mandatory MRA treatment in patients with HF of NYHA class III–IV, whereas the ‘less strict’ pattern did not (see Table 1). The true proportion of patients eligible for MRA treatment should lie within these two extremes. Diuretics were shown to alleviate the signs and symptoms of pulmonary and systemic venous congestion. Even if not strictly mandatory according to the guidelines, for example, in patients with HF of NYHA class I, the vast majority of individuals with HF receives diuretics [17, 21]. Thus, in addition to the therapies proven to reduce mortality, diuretics were considered in both patterns for all patients with HF of NYHA class II–IV. Data were analysed according to NYHA class, age group, sex, and whether hospitalization or death had occurred at any time during 2011.

## Results

### Total study population and subgroups

Of the 4,088,854 patients in the database sample, 3,132,337 had uninterrupted data available for the period between 1, January 2009 and 31, December 2013 and had not died before 1, January 2011. Approximately 4% of these patients, a total of 123,925, had two or more HF-related diagnostic codes recorded in 2011. This cohort was used in analyses of treatment patterns. Of these 123,925 patients, a subgroup of 26,368 individuals (21.3%) with a new diagnosis of HF was identified based on the absence of an HF-related diagnosis in the year before their first diagnosis in 2011. All analyses for the patient pathway relate to this subgroup with incident HF.

### Pathway of patients with newly diagnosed HF

**Fig. 1:**
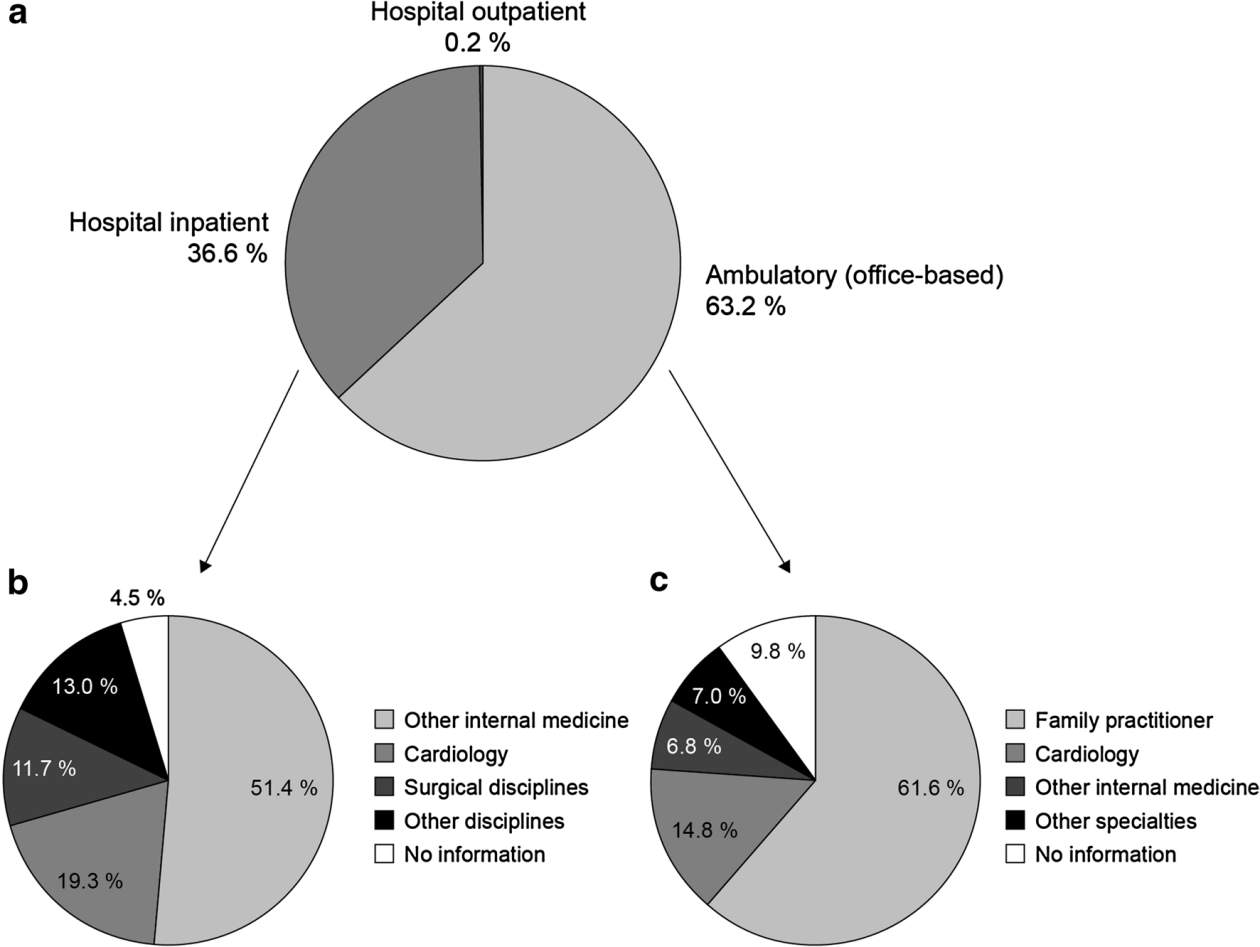
Proportion of new heart failure diagnoses made by physicians according to speciality within, **a** all settings combined (*N* = 26,368), **b** the hospital inpatient setting (*N* = 9653), and **c** the ambulatory (office-based) setting (*N* = 16,653)

Of the 26,368 patients with a new diagnosis of HF, the majority (63.2%) received their diagnosis in the ambulatory (i.e., office-based) setting, whereas about one-third (36.6%) of diagnoses were made in a hospital inpatient setting (Fig. 1). Very few patients were diagnosed with HF in a hospital outpatient setting (0.2%). Of diagnoses made in the ambulatory setting, the majority (61.6%) were made by family practitioners (comprising GPs and general internal specialists and practitioners). The second most common group of physicians diagnosing HF in the ambulatory setting was office-based cardiologists, who made 14.8% of diagnoses. In the hospital sector, almost three out of four patients were diagnosed with HF in departments of internal medicine, with the most frequent diagnosing unit being cardiology (19.3%).

**Fig. 2:**
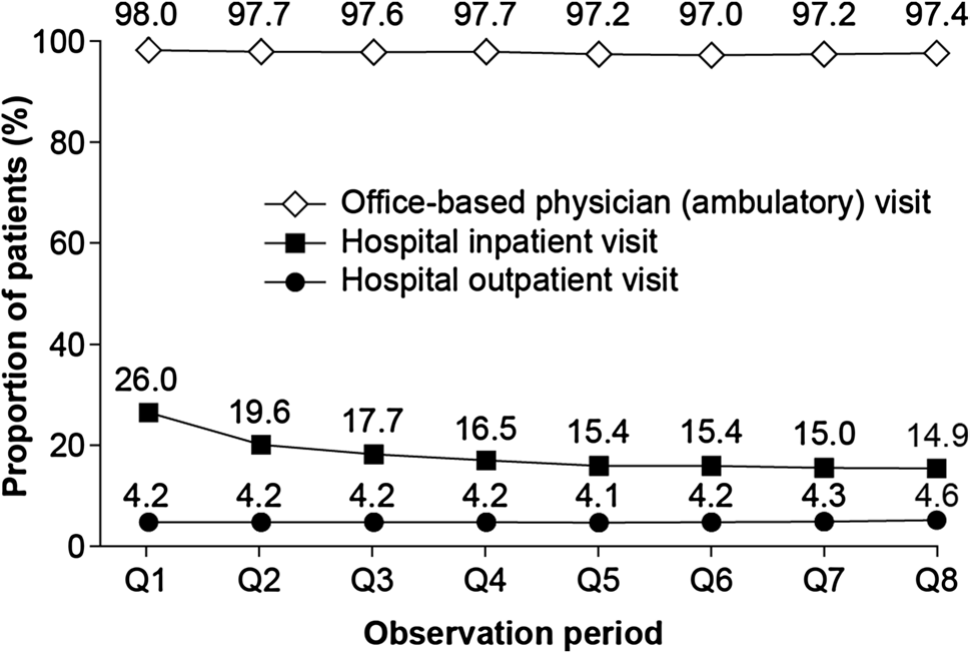
Proportion of patients with newly diagnosed heart failure who had at least one visit to an office-based physician (*N* = 24,641 in *Q1*), or to a hospital as an inpatient (*N* = 6541 in *Q1*) or outpatient (*N* = 1058 in *Q1*), in the two years after diagnosis. Proportions are relative to the number of patients alive in each quarter (*Q*)

Almost all (98.0%) patients had at least one visit to an office-based physician in the first quarter following their initial diagnosis of HF; this proportion remained similar for each quarter of the 2-year follow-up period (Fig. 2). In contrast, about one in four patients (26.0%) visited the hospital as an inpatient in the first quarter following initial diagnosis; this rate decreased to 14.9% over subsequent quarters of the 2-year period. Only a small percentage of patients (4.1–4.6%) visited hospital outpatient clinics in the two years following diagnosis.

**Fig. 3:**
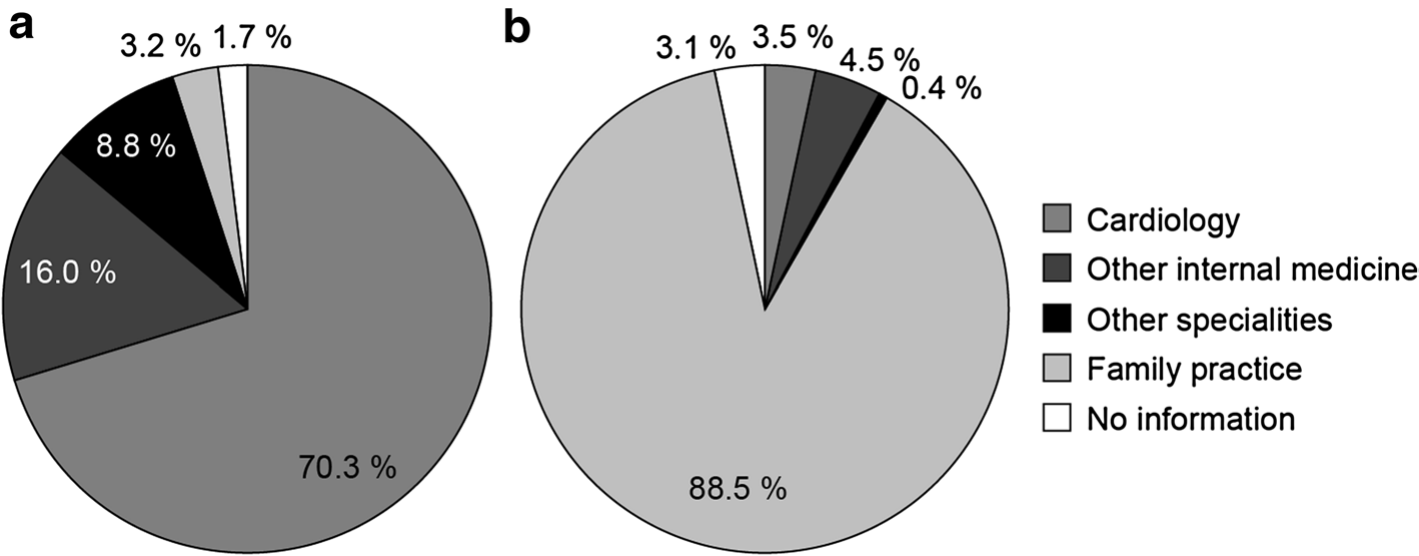
Proportions of heart failure-related activities and procedures carried out by physicians according to speciality within an office-based (ambulatory) setting in the two years after diagnosis in patients with newly diagnosed heart failure: a technical diagnostics (*N* = 28,704) and b treatment (*N* = 94,803)

**Fig. 4:**
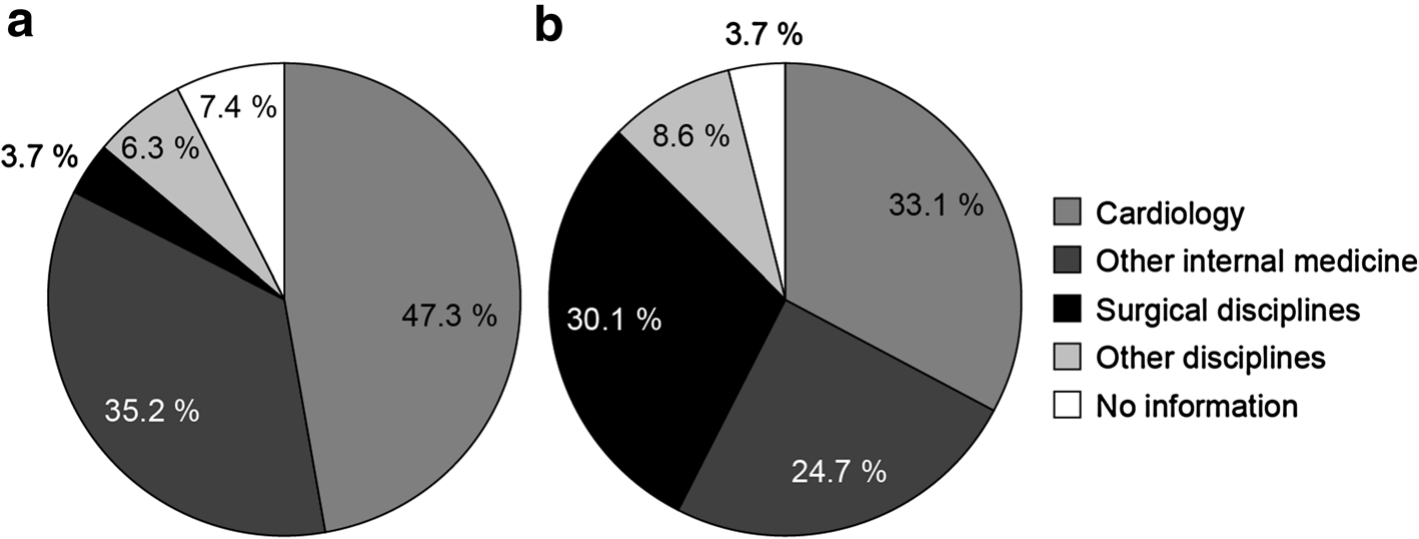
Proportions of heart failure-related activities and procedures carried out by physicians according to speciality within a hospital setting in the two years after diagnosis in patients with newly diagnosed heart failure: **a** technical diagnostics (*N* = 5873) and **b** treatment (*N* = 3061)

In an ambulatory setting, 86.3% of all HF-associated technical diagnostics were undertaken by specialists in internal medicine, most of whom (70.3%) had a background in cardiology (Fig. 3). The picture was similar in the hospital sector: 82.5% of all technical diagnostics were carried out in cardiology and other internal medicine departments (Fig. 4). The responsibility for treatment-related activities and procedures in the ambulatory sector following a new HF-related diagnosis fell primarily on family practitioners (88.5%) (Fig. 3). Only a small proportion of treatment-related activities and procedures were assigned to cardiologists (3.5%) and other internal medicine disciplines (4.5%) in the ambulatory sector, whereas in the hospital setting, the majority (57.8%) were carried out by the departments of internal medicine (33.1% cardiology; 24.7% other internal medicine disciplines), and almost one-third (30.1%) were performed by physicians from a surgical discipline (Fig. 4). Of the patients newly diagnosed with HF in 2011, 37% (9728) were assessed using a guideline-recommended echocardiographic procedure during the 2-year follow-up period. Our data focus on the most frequent EBM codes charged; it is, therefore, possible that an individual patient may have received additional tests during the follow-up period.

### Use of common guideline-recommended treatment regimens in all patients with HF

**Fig. 5:**
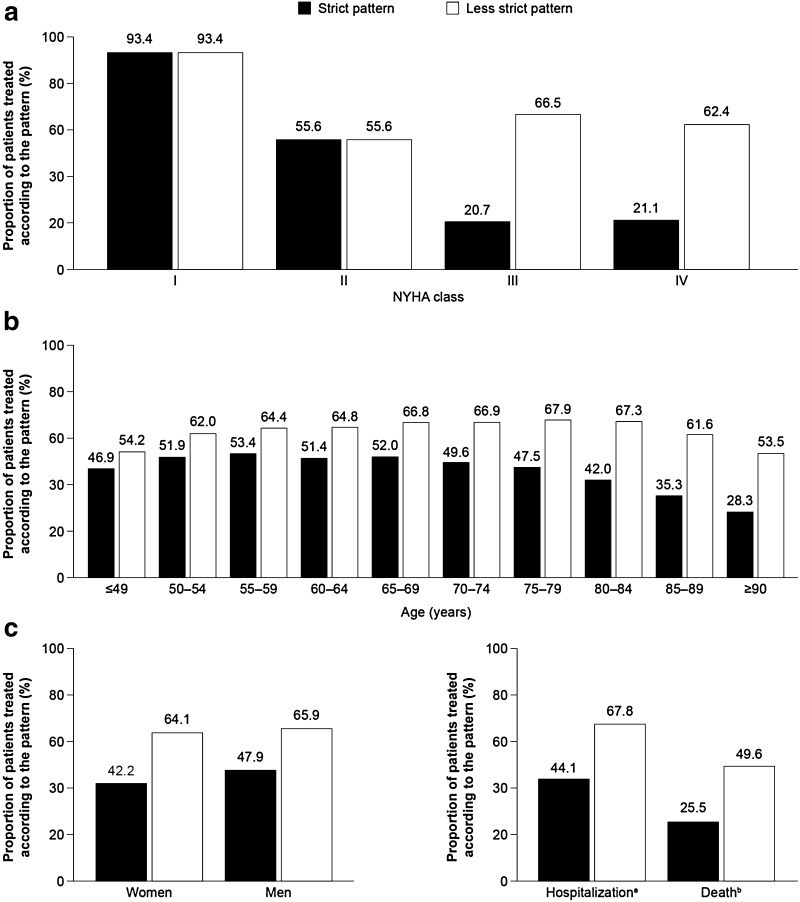
Application of the strict (*N* = 10,245) and less strict (*N* = 16,817) treatment regimens (see Table 1) in the two years after diagnosis in all patients with heart failure by: **a** NYHA class, **b** age group, and c sex, hospitalization, and death. *a* Similar proportions of patients were hospitalized whether they received a common treatment regimen according to the strict pattern or not (62.7 and 65.3%, respectively). *b* Mortality was lower in patients who received a common treatment regimen according to the strict pattern than in those who did not (6.7 vs. 16.2%, respectively), *NYHA* New York Heart Association

Of the 123,925 patients with two or more HF-related diagnostic codes recorded in 2011, one-fifth (20.9%, *N* = 25,863) had a specific NYHA class code associated with one or more of the HF-related diagnoses: 3518 (13.6%) were classified as having HF of NYHA class I, 10,992 (42.5%) as NYHA class II, 8,039 (31.1%) as NYHA class III, and 3314 (12.8%) as NYHA class IV. Of the 25,863 patients with HF with an assigned NYHA class, fewer than half (*N* = 11,669, 45.1%) received the strict treatment regimen (i.e., mandatory use of an MRA for patients with HF of NYHA class III–IV) in the first quarter following initial diagnosis. The proportion of patients receiving treatment according to the strict treatment pattern decreased with HF of increasing NYHA class (Fig. 5a) and older age, with just 28.3% of patients aged ≥90 years receiving such treatment (Fig. 5b**)**. More men than women received treatment consistent with the strict pattern (47.9 vs. 42.2%, respectively; Fig. 5c). In addition, 44.1% of patients who were hospitalized at any point during 2011, and 25.5% of patients who died, received treatment according to the strict pattern (Fig. 5c).

The proportion of patients treated according to the less strict treatment pattern (i.e., no mandatory use of an MRA for patients with HF of NYHA class III–IV) was greater than the proportion treated according to the strict pattern: 65.0 vs. 45.1%, respectively. These findings demonstrate the importance of MRA use driving the application of guideline-recommended treatment patterns in patients with HF of NYHA classes III–IV. The relationship between age and the likelihood of receiving such regimens was less pronounced than it was for the strict pattern; rates increased with age until the 75–79-year age bracket, but decreased with age in patients aged >80 years (Fig. 5b. Similar proportions of men and women received treatment that adhered to the less strict pattern (Fig. 5c).

## Discussion

This is the first large-scale, retrospective healthcare claims database study to report real-world patterns of HF treatment in Germany in 2009–2013, with a focus on the patient pathway and the use of treatment regimens linked to European and German guidelines in the 2 years after diagnosis of HF in 2011.

### Pathway of patients with newly diagnosed HF

The findings of our study demonstrate the pivotal role played by family practitioners as the primary point of contact in the initial diagnosis of HF and its treatment. Almost two-third of initial HF diagnoses were made in the ambulatory setting, and of these, almost two-third were made by a family practitioner. In addition, unlike hospital visits, almost all patients visited an office-based physician at least once every quarter in the two years after the first diagnosis of HF. The responsibility for treatment-related activities and procedures also appears to lie primarily with family practitioners in the ambulatory setting, with cardiologists and other internal medicine specialists involved to a lesser degree. The element of care in which family practitioners appeared to be less involved was HF-related technical diagnostics, for which internal medicine specialists (including cardiologists) in both the ambulatory and the hospital settings took responsibility. Hence, our findings confirm two important notions: (1) physicians from various specialities and healthcare sectors are involved in the treatment of patients with HF, underlining the need for structured rules of communication and interaction between these parties; (2) the primary contact for most patients with HF is their family practitioner who acts as gatekeeper for the diagnosis and treatment of HF; such a role, however, mandates close cooperation with specialists in internal medicine required for technical diagnostics, in particular echocardiography.

### Use of common guideline-recommended treatment regimens in all patients with HF

Overall, in 2011, the treatment of patients with HF and an assigned NYHA class complied with the strict treatment pattern in 45.1% of individuals. Adherence to this regimen decreased with older age and severity of disease: approximately one-fifth of all patients with HF assigned NYHA classes III–IV, and one-quarter of patients aged ≥90 years, were treated according to this common guideline-recommended pattern. As expected, the application of the less strict version of the treatment regimen was greater than that of the strict regimen across HF of NYHA classes III–IV. These findings were expected because the less strict pattern does not mandate the use of an MRA in patients with HF of NYHA class III–IV who have contraindications to the drug class. Therefore, the results for the less strict pattern are likely to overestimate the proportion of patients receiving common guideline-recommended treatment regimens, whereas those for the strict pattern are likely to underestimate the proportion.

Of all the patients who died, just one-quarter were found to have received treatment according to the strict pattern; mortality was lower among individuals treated according to this pattern than among those who were not. Similar findings regarding physician’s adherence to guideline-recommended treatment were reported in an international study of 6669 outpatients with HFreF, where poor adherence was associated with significantly higher overall mortality and heart failure mortality [22]. However, in our study, the proportion of elderly patients (aged >80 years) was greater in the subgroup of patients who were not receiving treatment according to the strict pattern, which may confound the interpretation of higher mortality in this group.

The observation that the number of patients receiving treatment regimens consistent with guidelines declines with age has been documented previously [17], and can perhaps be explained by the fact that older patients often have several comorbidities, and therefore, are likely to be receiving additional medication. Elderly patients frequently have impaired renal function and may, therefore, be less tolerant of aggressive treatment regimens and more susceptible to drug–drug interactions or drug-related adverse events. Therefore, physicians may prioritize the overall health of their patient over rigorous compliance with guidelines; in particular, physicians may be less willing to prescribe MRAs in patients with reduced renal function than in those with normal renal function [2, 17, 23]. Moreover, the risk of falls in elderly patients is a particular concern, and this is increased in individuals with low blood pressure; therefore, physicians may also be less willing to prescribe blood pressure-reducing medications extensively, such as those used for the treatment of HF. The decline in guideline-consistent therapy with increased severity of HF may be explained by the increased complexity of the recommended pharmacological regimen. Furthermore, patients with more severe disease are more likely to be elderly, and therefore, are more likely to have the aforementioned comorbidities and complexities.

It should be emphasized that the treatment regimens analysed here are recommended only for HFrEF. It was assumed that approximately half of the patients in this analysis would have this type of HF [15]; the other half would have HFpEF. The guidelines focus on the treatment of underlying diseases in patients with HFpEF; hence, 100% usage of these treatment regimens was not expected. As treatment for individuals with HFpEF is typically less aggressive than treatment for patients with HFrEF [24], the proportions treated according to common guideline-based treatment patterns would be expected to be higher in a patient group including only individuals with HFrEF; values are, therefore, likely to underestimate the use of these patterns among patients with HFrEF. Indeed, the results of other recent studies of adherence to ESC guidelines for management of HF in Germany suggest that adherence is high [2, 17, 18], with rates of more than 80% in some cases [2]. However, these studies analysed treatment patterns in patients with HFrEF only, rather than a mixed group of patients, so adherence rates would be expected to be higher than those in the current study. These studies were also relatively small, and examined a small range of settings, so the proportion of patients treated by specialists would have differed from that found in the current study. It should also be noted that rigorous adherence to treatment guidelines is not always appropriate. Treatment steps are dependent on whether the patient’s condition is stable under current therapy. In the present study, this was not easily discernible from the data available, with change in NYHA class used as an approximate measure. Detailed information on comorbidities and other factors that may be contraindications for medication was also unavailable. When such factors are considered, the use of guideline-consistent treatment increases further [21].

### Clinical implications

How can HF be optimally managed in the German healthcare system? What is the best way to address the intersectoral information and care gaps? Multidisciplinary care programmes and nurse-facilitated care are among the strongest recommendations in all HF guidelines since 2008 (class IA indication). There is evidence from randomized controlled trials that involvement of dedicated staff improves mortality risk, long-term morbidity, quality of life, and left ventricular remodelling [25]. There is further evidence that a sizeable part of this effect is due to up-titration in the multi-faceted treatment plans of this complex patient population [26]. However, in the German healthcare system, such an approach is not reimbursed, and therefore, does not form part of general practice. The currently planned HF Disease Management Programme should incorporate these well-founded recommendations to become effective on a larger scale.

### Strengths and limitations

Strengths of this study include the large sample size used—at least an order of magnitude larger than that in recent similar studies in Germany [2, 17, 18]—and the fact that data were taken from a well-validated database [19]. In addition, in contrast to many other observational studies, the patients in our study are likely to be highly representative of the German population. This is because other non-interventional or registry studies often analyse data obtained from a limited number of sites, which are often in the tertiary care setting. Our study analysed data from a range of healthcare settings, including hospitals and outpatient care centres, as well as from the ambulatory setting.

This study is subject to limitations typical of observational studies, relating to the quality and representativeness of the underlying data. The HRI database captures only individuals with SHI and not those with private health insurance. There is also a reliance on the accuracy of diagnoses and coding. Documentation of NYHA functional class was available in only a subset of the population, and the nature of the data set does not allow statements to be made about the generalizability of our findings to patients in whom NYHA class was not documented.

## Conclusions

This retrospective claims database study of the treatment of patients with HF in Germany demonstrates the key role of the family practitioner in the initial diagnosis of patients. Close collaboration and communication between healthcare professionals in all specialties and sectors is essential for providing optimal care for these patients. Further studies are needed to quantify the effectiveness of patient pathways in terms of costs and patient outcomes. For many patients, treatment patterns were consistent with regimens recommended by European and German guidelines; however, there seems to be scope for greater consistency among older patients and in individuals with advanced disease. Specific studies considering HFrEF and HFpEF status, as well as other clinical factors, are needed to further our understanding of the importance of improving the adherence of drug prescription in German clinical practice to treatment regimens advocated by guidelines.

## Electronic supplementary material

Below is the link to the electronic supplementary material.

Supplementary material 1 (PDF 147 kb)
