# Supplementary material for: Treatment of chronic heart failure in Germany: a retrospective database study
Source: Clin Res Cardiol. 2017 Jul 26;106(11):923–32. doi: 10.1007/s00392-017-1138-6 (PMC5655600; doi:10.1007/s00392-017-1138-6)
Supplement: Supplementary file 1 — Supplementary material 1 (PDF 147 kb) [file 392_2017_1138_MOESM1_ESM.pdf]

Electronic supplementary material

Treatment of chronic heart failure in Germany: a retrospective database study

Clinical Research in Cardiology

Stefan Störk • Renate Handrock • Josephine Jacob • Jochen Walker • Frederico Calado • Raquel Lahoz Stephan Hupfer • Sven Klebs

Corresponding author:

Professor Stefan Störk

Comprehensive Heart Failure Centre Würzburg and Department of Internal Medicine I, University and University Hospital Würzburg, Würzburg, Germany

e-mail address: Stoerk_S@ukw.de

Online Resource 1 Cardiovascular medication substance groups included in the analysis

ATC code	Designation in ATC/DDD Index 2013 (German version)
C01A	Cardiac glycosides
C01AA04	Digitoxin
C01AA05	Digoxin
C01E	Other cardiac preparations
C01EB17	Ivabradine
C03A	Low-ceiling diuretics, thiazides
C03B	Low-ceiling diuretics, excl. thiazides
C03C	High-ceiling diuretics
C03D	Potassium-sparing diuretics
C03DA01	Spironolactone
C03DA04	Eplerenone
C03E	Diuretics and potassium-sparing agents, combination
C03EC01	Spironolactone and low-ceiling diuretics
C03EC21	Spironolactone and hydrochlorothiazides
C03EC41	Spironolactone and bendroflumethiazide
C03ED01	Spironolactone and high-ceiling diuretics
C07A	β-blocking agents, plain
C07AB07	Bisoprolol
C07AB12	Nebivolol
C07AB02	Metoprolol
C07AB52	Metoprolol, combination
C07AG02	Carvedilol
C07B	β-blocking agents and thiazides
C07BB02	Metoprolol and thiazides
C07BB07	Bisoprolol and thiazides
C07BB12	Nebivolol and thiazides
C07BG02	Carvedilol and thiazides
C07C	β-blocking agents and other diuretics
C07CB02	Metoprolol and other diuretics
C07D	β-blocking agents, thiazides and other diuretics
C07F	β-blocking agents and other antihypertensives
C07FB02	Metoprolol and other antihypertensives
C07FB07	Bisoprolol and other antihypertensives
C07FB22	Metoprolol and nifedipine
C07FB24	Metoprolol and felodipine
C08C	Calcium-channel blockers
C08CA01	Amlodipine
C08CA02	Felodipine
C09A	ACE inhibitors, plain
C09AA03	Lisinopril
C09AA05	Ramipril
C09AA02	Enalapril
C09AA01	Captopril
C09B	ACE inhibitors, combination
C09BA01	Captopril and diuretics
C09BA05	Ramipril and diuretics
C09BA25	Ramipril and hydrochlorothiazides
C09BA02	Enalapril and diuretics
C09BA03	Lisinopril and diuretics
C09BB03	Lisinopril and amlodipine

C09BB05	Ramipril and felodipine
C09C	Angiotensin II antagonists, plain
C09CA01	Losartan
C09CA03	Valsartan
C09CA06	Candesartan
C09D	Angiotensin II antagonists, combination
C09DA01	Losartan and diuretics
C09DA03	Valsartan and diuretics
C09DA06	Candesartan and diuretics
C09DB01	Valsartan and amlodipine
C09DB06	Losartan and amlodipine
C09DX01	Valsartan, hydrochlorothiazides, and amlodipine

ACE angiotensin-converting enzyme, *ATC* Anatomical Therapeutic Chemical, *DDD* defined daily dose
